# Dichloroacetate affects proliferation but not apoptosis in canine mammary cell lines

**DOI:** 10.1371/journal.pone.0178744

**Published:** 2017-06-07

**Authors:** Tatjana P. Harting, Mandy Stubbendorff, Susanne C. Hammer, Patrik Schadzek, Anaclet Ngezahayo, Hugo Murua Escobar, Ingo Nolte

**Affiliations:** 1 Small Animal Clinic, University of Veterinary Medicine Hannover, Foundation, Hannover, Germany; 2 Division of Medicine Clinic III, Hematology, Oncology and Palliative Medicine, University of Rostock, Rostock, Germany; 3 Evotec AG, Hamburg, Germany; 4 Institute of Biophysics, Leibniz University, Hannover, Germany; Columbia University, UNITED STATES

## Abstract

Targeting mitochondrial energy metabolism is a novel approach in cancer research and can be traced back to the description of the Warburg effect. Dichloroacetate, a controversially discussed subject of many studies in cancer research, is a pyruvate dehydrogenase kinase inhibitor. Dichloroacetate causes metabolic changes in cancerous glycolysis towards oxidative phosphorylation via indirect activation of pyruvate dehydrogenase in mitochondria. Canine mammary cancer is frequently diagnosed but after therapy prognosis still remains poor. In this study, canine mammary carcinoma, adenoma and non-neoplastic mammary gland cell lines were treated using 10 mM Dichloroacetate. The effect on cell number, lactate release and PDH expression and cell respiration was investigated. Further, the effect on apoptosis and several apoptotic proteins, proliferation, and microRNA expression was evaluated. Dichloroacetate was found to reduce cell proliferation without inducing apoptosis in all examined cell lines.

## Introduction

Mammary tumors are the most frequent neoplasia in intact female dogs and often accompanied with high recurrence and metastasis rates [1]. In veterinary medicine as well as human medicine a surgical intervention is indicated often with subsequent chemotherapy [1]. In advanced tumor disease the prognosis still remains poor [1] therefore new alternatives for chemotherapy have to be investigated.

The Warburg effect was characterized in the early 1920s by Otto Warburg and describes the metabolic energy production of most cancer cells which rely on aerobic glycolysis in presence of oxygen [2, 3]. Hypoxia in early cancer transformation results in expression of hypoxia inducible factor 1 alpha (HIF-1α) activating pyruvate dehydrogenase kinase (PDK), a pyruvate dehydrogenase (PDH) inhibiting enzyme [4]. PDH inhibition prevents incorporation of pyruvate in mitochondria and is related with cytoplasmic metabolization of pyruvate to lactate [4]. Compensation of negative energy output during glycolysis occurs with increased expression of glycose transporters induced by HIF-1α [5]. Glycolysis contains several advantages for cancer progression such as lactic acidosis allowing tumor growth due to damage of extracellular matrix and increased cell mobility [6]. Decreased cell respiration leads to lower production of reactive oxygen species (ROS) in mitochondria as well as decreased DNA damage and enables apoptosis resistance [7, 8]. The glycolytic feature of cancer cells might offer a selective therapeutic target sparing treatment of non-cancerous cells [3].

Dichloroacetate (DCA) is a pyruvate dehydrogenase kinase inhibitor [9] and thus enhances the flux of pyruvate into the mitochondria by indirect activation of pyruvate dehydrogenase [10]. By reason of occurring glycolytic profile in cancer and penetration of most tissues after oral administration [10, 11], DCA appears to be a proficient strategic therapeutic target in oncology [11]. The last decades, DCA was used as lactate lowering drug in human with congenital mitochondrial dysfunction in phase III studies [12, 13] and became a controversially discussed subject in cancer research. Michelakis et al. found that DCA normalized mitochondrial function and decreased cancer growth *in vitro* and pointed out that non-cancerous cells were not affected [14]. Dunbar et al. reported that DCA was well tolerated and feasible in a phase I trial in patients suffering from recurrent glioblastomas [15] but in contrast, another study had to be cancelled due to severe neuropathies [16]. DCA in human mammary tumors showed inconsistent results. Feuerecker et al. reported higher viability and proliferation in human SrBr3 cells after DCA treatment [17] whereas Sun et al. determined inhibited cell growth in several mammary cancer cell lines [18]. Higher apoptotic resistance [19] as well as increased mitochondrial depolarization was reported in human MCF-7 cells [11].

In previous studies we investigated the effect of DCA on canine prostate adenocarcinomas and transitional cell carcinomas in vitro [20] and found that DCA has anti-proliferative effects on canine prostate and bladder tissue derived cell lines. Until now, there is no data available analyzing the effects of DCA on canine mammary tumors. DCA seems to be well tolerated in dogs with lactic acidosis [21] and other studies concerning pharmacokinetic effects [22, 23].

For evaluation of anticancer drug efficacy in preclinical experiments cell lines represent important *in vitro* models to gain more information of cancer independent sensitivity [24–26]. In this study several cell lines derived from canine mammary tissue were used in order to evaluate DCA efficiency.

This is the first study evaluating the effect of 10 mM DCA on canine mammary carcinoma as well as canine mammary adenoma cell lines in comparison to a non-cancerous mammary gland derived cell line and a non-treated control. Therefore, the influence on cell counts, viability, apoptosis, mitochondrial respiration and proliferation was examined. Furthermore, the expression of microRNA involved in proliferation and apoptosis was determined.

## Materials and methods

### Cell lines

Four cell lines derived from different mammary tissues were used for experiments. MTH53A (non-neoplastic mammary gland), MTH52C (mammary carcinoma) and ZMTH3 (mammary adenoma) were transfected with SV-40 and routinely maintained in the Small Animal Clinic, University of Veterinary Medicine, Hannover, Germany. SV-40 transfection was performed to achieve immortalization as primary cultures derived from mammary tissue (MTH53A, MTH52C and ZMTH3) showed limited proliferation capacity. DT14/06T (mammary carcinoma) was established by continuous cultivation in the Small Animal Clinic, University of Veterinary Medicine Hannover, Germany. The cell lines were classified after pathohistological examination of the primary tissue. All tissues used for cell line establishment were collected as part of routine mastectomy and with the owner’s permission. Therefore according to German standards, ethical approval was not required.

### Cell culture

All cell lines were routinely maintained in 25 cm^2^ culture flasks (TPP, Faust Lab Science, Klettgau, Gemany) with medium 199 (Gibco^TM^, Thermofisher Scientific, Darmstadt, Germany) containing 10% fetal calf serum (HyClone®, Thermofisher Scientific), 2% penicillin-streptomycin (Biochrom, Berlin, Germany) and incubated at 37°C and 5% CO_2_ in humidified atmosphere. At 85% confluency cells were trypsinized with TrypLE^TM^ Express (Gibco^TM^, Thermofisher Scientific) and were splitted 1:2.

### DCA application

Cells used in experiments were exposed to 10 mM DCA over 48 hours and cultured with 10 ml medium in 75 cm^2^ flasks (TPP, Faust Lab Science). DCA was diluted in deionized water and filter-sterilized after pH modulation to 7.4 with NaOH. All cell lines were cultured without DCA and used as non-treated control respectively. The concentration of 10 mM DCA was chosen in order to allow comparability with previous human [27] and canine [20] *in vitro* studies. Furthermore, this dosage is applied in an explorative study setting.

### Cell counting

After washing with PBS to remove dead cells and trypsinization with TrypLE^TM^ Express (Gibco^TM^, Thermofisher Scientific) cell number was counted with an automated Cellometer^TM^ Auto T4 (Nexcelom Bioscience, Lawrence, MA, USA) and compared with untreated control. Cells were washed with PBS, centrifuged at 1000 rpm for 10 minutes (Biochrom) and stored at -80°C for further examinations (quantitative RT-PCR and protein analysis).

### Lactate levels

Lactate release in media was performed by colorimetric determination with Cobas® C311 (Hitachi, Tokio, Japan). Therefore growth media from cell culture was centrifuged at 1000 rpm for 10 minutes to remove floating cells and debris and 1.3 ml were removed to a sodium fluoride vessel (Sarstedt, Nümbrecht, Germany). To eliminate influences of phenol red and fetal calf serum, medium was used as negative control and deducted from measurements. Further the lactate content was normalized to intracellular protein concentration to prevent influences of cell numbers and volume. Protein concentration was assessed with Pierce^TM^ BCA Assay (Thermofisher Scientific) as described in manufacturer´s instructions.

### Metabolic activity

Depending on each cell line 3000–6000 cells were seeded in a 96-well-plate (Falcon, Corning, Amsterdam, The Netherlands) with 200 μl medium 199, 10% fetal calf serum, 2% penicillin-streptomycin, 10 mM DCA and incubated at 37°C and 5% humidified CO_2_. Measurements were performed every 24 hours for four days. Therefore medium was replaced and 20 μl MTT (CellTiter96® Aqueous One Solution Assay, Promega, Mannheim, Germany) was added and incubated for two hours at 37°C in the dark. Absorbance was determined with a Synergy2 plate reader (BioTek, Bad Friedrichshall, Germany) and data were analyzed with Gen5^TM^ 1.11 Software (BioTek). Absorbance was reduced by media negative control and in addition normalized to non-treated new seeded cells.

### Flow cytometry

Apoptosis and quantity of dead cells was analyzed with Annexin FITC and Sytox labeling (Annexin V-FITC Detection Kit Plus, PromoCell, Heidelberg, Germany). Therefore 10^5^ cells were cultured in a 6-well-plate (TPP, Faust Lab Science) as described above. After 48 hours following 10 mM DCA exposure, cells were trypsinized and centrifuged together with supernatant containing non-adherent and dead cells at 1000 rpm for 6 minutes. Cell pellet was resuspended in 500 μl assay buffer and staining was performed as described in manufacturer’s protocol. 10^4^ events were counted with BD FACScalibur^TM^ (BD Biosciences, Heidelberg, Germany) and CellQuest^TM^ Pro 6.0 software (BD Biosciences). Annexin and Sytox were detected in FL-1. Data analysis was performed with FlowJo Version 10.0.8r1 (FlowJo, Ashland, OR, USA). Gates were determined based on positive controls (cells permeabilized with Saponin) and negative controls of each cell line (non-treated viable cells).

### RNA isolation and quantitative RT-PCR

Isolation of total RNA was performed with 10^6^ cells using the NucleoSpin Small RNA Kit (Macherey Nagel, Düren, Germany) as described in manufacturer’s protocol. 35 ng total RNA was used for cDNA synthesis using Taqman® MicroRNA Reverse Transkription Kit (Applied Biosystems^TM^, Thermofisher Scientific) according to manufacturer´s instructions. Following cycle conditions were used: 30 minutes 16°C, 30 minutes 42°C, 5 minutes 85°C. Relative quantification of microRNA expression of treated cells in comparison to untreated control was performed with Eppendorff realplex^4^ Cycler (Eppendorf, Wesseling-Berzdorf, Germany) using 1.33 μl cDNA in a total volume of 20 μl containing TaqMan® Universal Master Mix NoAmpErase® UNG (Applied Biosystems^TM^, Thermofisher Scientific) and TaqMan® MicroRNA assays for Mir141 (ID 245445_mat), Mir145 (ID 002278), Mir375 (ID 000564) purchased from Thermofisher Scientific. Procedure was maintained as manufacturer´s protocol conducting following conditions: 95°C for 10 minutes subsequently 40 cycles of 95°C for 15 seconds and 60°C for 60 seconds. Data were normalized to the reference gene RNU6B (ID 001093) and analysis was performed using Rest2009 (Qiagen, Hilden, Germany). Samples with Ct-values >35 were excluded from analysis.

### Luminex magnetic bead analysis

Samples for protein expression analysis were prepared as specified in manufacturer´s protocol and measurements were performed with xMAP® Luminex Bead Technology using a Luminex 200^TM^ instrument (Luminex Corporation, Hertogenbosch, The Netherlands) and processed with xPONENT 3.1 software (Luminex Corporation). Additionally, samples for PDH measurements were filtered with centrifugal ultrafree filter units with a pore size of 0.65 μm (Merck Millipore) at 7000 rpm for 4 minutes. Values with MFI < background MFI + 2 x standard deviation were excluded from analysis. Quantitation of survivin was performed with ProcartaPlex Human Survivin Simplex Kit (eBioscience, Frankfurt am Main, Germany) using cell culture supernatant as described in manufacturer´s protocol. Survivin values were normalized to protein concentration (Pierce BCA Assay, Thermofisher Scientific). Total PDH, PDH-P and apoptotic proteins (BAD and JNK) were analyzed with multiplex assays from Merck Millipore (Multi-species PDH Complex Magnetic Bead Panel and 7-Plex Early Apoptosis Magnetic Bead Kit, Darmstadt, Germany). For protein evaluation a total protein amount of 10 ng (PDH-P) or rather 25 ng (apoptotic proteins JNK and BAD) was used, which was determined with Pierce BCA Assay according to manufacturer’s instruction (Thermofisher Scientific).

### Mitochondrial ROS production

Mitochondrial ROS production as indirect marker for mitochondrial activity was determined by staining of mitochondrial derived ROS using 4 μM MitoSox (Invitrogen, Thermofisher Scientific) for 15 minutes. Cells were grown on 8-well μ-dishes (Ibidi, Martinsried, Germany) treated with 10 mM DCA for 48 hours. After fixation with 4% paraformaldehyde, μ-slides were washed with Hank´s Balanced Salt Solution (HBSS) containing calcium and magnesium. After staining cell nuclei were labeled with DAPI (dilution of 1:1000, Sigma Aldrich GmbH) for 5 minutes. Fluorescence imaging was performed with an inverted confocal laser scanning microscope (Eclipse TE2000-E, Nikon, Düsseldorf, Germany) using a 60x water immersion objective (Nikon). Images were taken with EZ-C1 1.80 software (Nikon). The excitation occurred with a diode laser at 408 nm (DAPI) and with a helium/neon laser at 543 nm (MitoSox). Total cell fluorescence of MitoSox deducting background was analyzed with ImageJ and normalized to cell counts.

### Mitochondrial ATP-synthesis

Mitochondrial respiration was directly shown by measuring cellular ATP synthesis. The cell lines were seeded (5000 cells/well) as triplicate in a 96-opaque-well plate (Greiner Bio-One) with 100 μl medium 199, 10% fetal calf serum, 2% streptomycin-penicillin, 10 mM DCA and incubated at 37°C humidified CO_2_. After 48 hours incubation 100 μl ATP-solution (CellTiter-Glo® 2.0 Assay, Promega) was added. Following 10 minutes incubation at room temperature, luminescence measurement was performed with Synergy2 Plate Reader (BioTek). In order to normalize the data with the respective cell numbers, cells were counted in a simultaneously performed experiment by flow cytometry (MACSQuant Analyzer 10, Miltenyi Biotek, Bergisch Gladbach, Germany).

### Immunofluorescence staining of Ki67 and TUNEL

Cultivation, fixation and washing were performed as described above. After permeabilization using 0.2% Triton X-100 for 20 minutes, cells were exposed overnight to a canine specific rabbit-polyclonal Ki67 antibody (dilution of 1:150; Life Technologies, Thermofisher Scientific). For labeling, a monoclonal anti-rabbit Alexa Fluor® 555 antibody (Cell Signaling Technology, Leiden, The Netherlands) was incubated for 1 hour (1:250) and cells were counterstained with DAPI (1:1000) for 5 minutes. Fluorescence imaging protocol was the same as described above. Total cell fluorescence was established as described above. For TUNEL staining Apoptag Fluorescein Direct Kit (Merck Millipore) was performed as manufacturer´s instructions. The excitation occurred with an argonlaser at 488 nm and imaging was performed as described above. The amount of TUNEL positive cells was evaluated.

### Western blot

Protein expression analysis was performed with Immunoblotting (Western Blot) as described in detail by Wagner et al. [28]. The protein concentration was determined using Pierce BCA Assay (Thermofisher Scientific) and for electrophoresis 60 μg protein of each sample was used. The PVDF membrane was incubated overnight at 4°C with a canine specific polyclonal primary antibody anti-rabbit anti-PDK-1 (1:1000, ABIN2499649, antibodies-online, Aachen, Germany) or a canine specific polyclonal anti-rabbit anti-PDK-3 antibody (1:500, ABIN278250, antibodies-online). Afterwards, the PVDF membrane was incubated with a secondary antibody anti-rabbit IgG, AP Conjugate for 2 hours (Promega, Mannheim, Germany). A canine specific monoclonal anti-mouse anti-GAPDH antibody (Abcam, Cambridge, United Kingdom) was used as loading control.

### Statistical analysis

Statistical analysis of data was performed with SAS software 7.1 (SAS Institute Inc., Cary, NC, USA). For comparison of two means, two-tailed t-test was used. The confidence value was set to 5% (p<0.05) and was considered statistically significant.

## Results

### DCA reduces cell number

**Fig 1:**
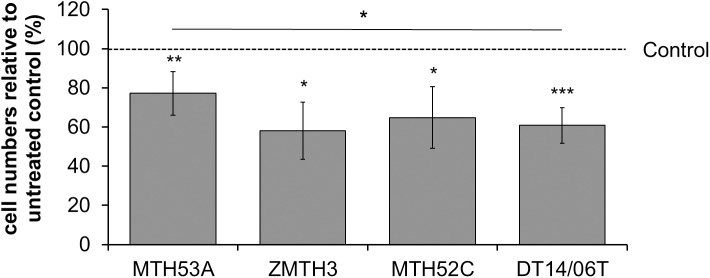
Influence of 10 mM DCA on mammary cell lines after 48 hours. Statistical significant reduction of cell number was observed in all cell lines. Significant difference in cell number between MTH53A and mammary carcinoma DT14/06T was observed. Data is shown as mean ± standard deviation (SD), n≥3 and are presented as relative cell numbers in comparison to the respective corresponding untreated control (%). Control values were set to 100%. Statistical analysis was performed with two-tailed t-test, *p<0.05, **p<0.01, ***p<0.001.

Following 48 hours DCA treatment (10 mM) the cell numbers of both mammary carcinoma cell lines MTH52C (p = 0.0171) and DT14/06T (p<0.0001) decreased significantly in comparison to non-treated control. A comparable significant effect was observed in the mammary adenoma cell line ZMTH3 (p = 0.0131) and could also be constituted in the non-cancerous mammary gland derived cell line MTH53A (p = 0.0039). Although MTH53A showed decreasing cell numbers, a statistically significant difference between the mammary carcinoma cell line DT14/06T (p = 0.0140) and the non-cancerous cell line MTH53A was present. No difference was observed between MTH53A and the adenoma derived cell line ZMTH3 or the mammary carcinoma derived cell line MTH52C (Fig 1).

### DCA affects proliferation

**Fig 2:**
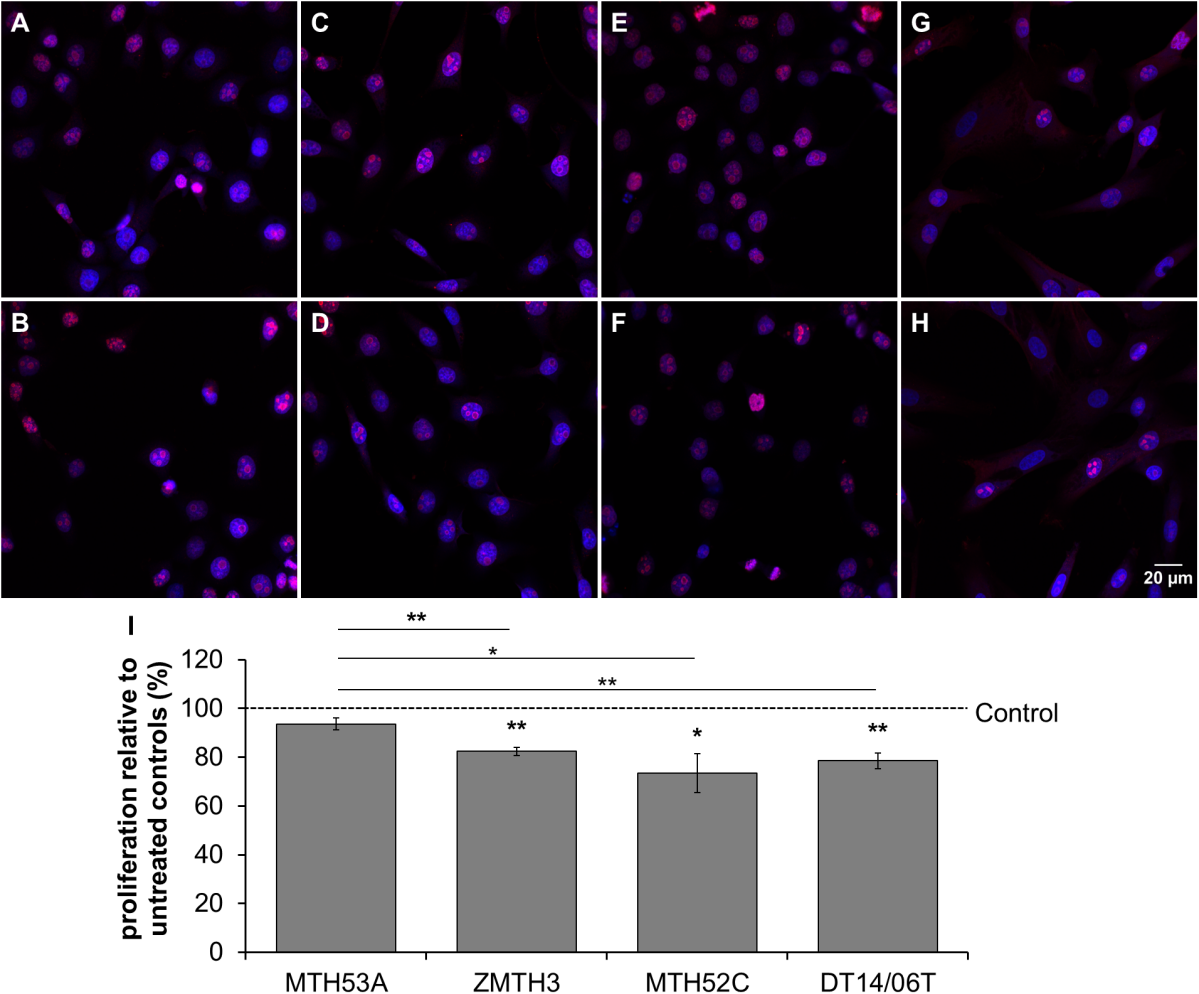
Impact of 10 mM DCA on proliferation of mammary cell lines after 48 hours determined with fluorescence microscopy. (A-H) Images showing fluorescence of Ki67 (red) and DAPI (blue). In comparison to non-treated control (A,C,E,G) amount of Ki67 is reduced in all DCA treated cell lines (B,D,F,H) except in MTH53A (B). Statistical significant reduction of Ki67 was observed in ZMTH3, MTH52C and DT14/06T in comparison to non-treated control and non-neoplastic tissue derived cell line MTH53A. No significant decrease in proliferation could be determined in within neoplastic cell lines (I). Data are shown as mean ± SD, n≥3 and are presented as relative proliferation in comparison to untreated control (%). Control was set to 100%. Statistical analysis was performed with two-tailed t-test; *p<0.05, **p<0.01, ***p<0.001. (A) MTH53A control; (B) MTH53A+DCA; (C) ZMTH3 control; (D) ZMTH3+DCA; (E) MTH52C control; (F) MTH52C+DCA; (G) DT14/06T control; (H) DT14/06T+DCA; (I) relative proliferation.

The proliferation, assessed by detection of the proliferation marker Ki67, decreased significantly in all mammary cell lines with exception of the non-neoplastic mammary gland derived cell line MTH53A in comparison to non-treated control and further a significant decrease in proliferation can be observed within comparison of MTH53A and the other cell lines. No difference was assessed between neoplastic cell lines (Fig 2).

### Decreased lactate release after DCA treatment

**Fig 3:**
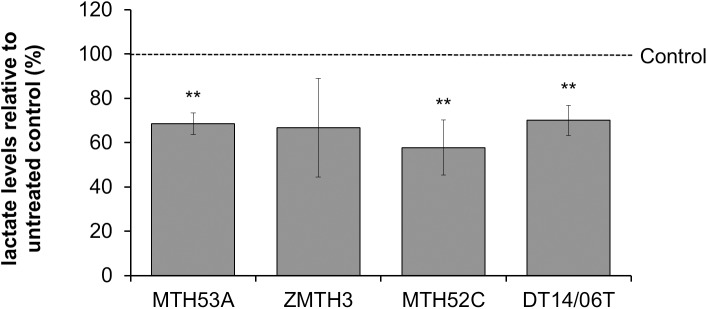
Effect of 10 mM DCA on lactate release of mammary cell lines after 48 hours. In comparison to untreated control significant reduction of lactate production was proved in all cell lines except the benign mammary adenoma (ZMTH3). No significant difference was evaluated between MTH53A and the other cell lines. Data are shown as mean ± SD, n≥3 and are presented as relative lactate content in comparison to untreated control (%). Control was set to 100%. Statistical analysis was performed with two-tailed t-test, *p<0.05, **p<0.01, ***p<0.001.

In comparison to negative control DCA reduced the lactate release into the media in all examined cell lines except in the benign mammary adenoma cell line ZMTH3 (p = 0.1218). No statistical difference was observed within non-cancerous and neoplastic cell lines (Fig 3).

### DCA did not alter PDK expression

**Fig 4:**
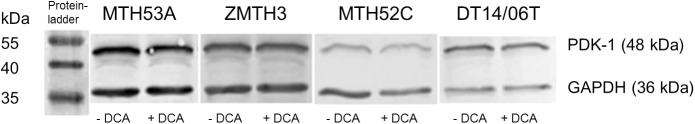
Effect of 10 mM DCA on PDK-1 expression after 48 hours. Western Blot analysis of PDK-1 expression in MTH53A, MTH52C, ZMTH3 and DT14/06T. All cell lines showed positivity for PDK-1 and GAPDH but no changes in PDK-1 expression was detectable between untreated and DCA exposed cells in any of the evaluated cell lines. GAPDH was used as loading control.

In order to assess the effect of DCA on PDK expression, protein analysis of PDK-1 and PDK-3 was performed. As shown in Fig 4 PDK-1 expression was visible in all examined cell lines (untreated and treated) at 48 kDa. In comparison to untreated control no changes could be determined. The protein PDK-3 (47 kDa) was not detectable in all cell lines (supplementary files, S2 Fig).

### Decreased PDH phosphorylation after DCA treatment

**Fig 5:**
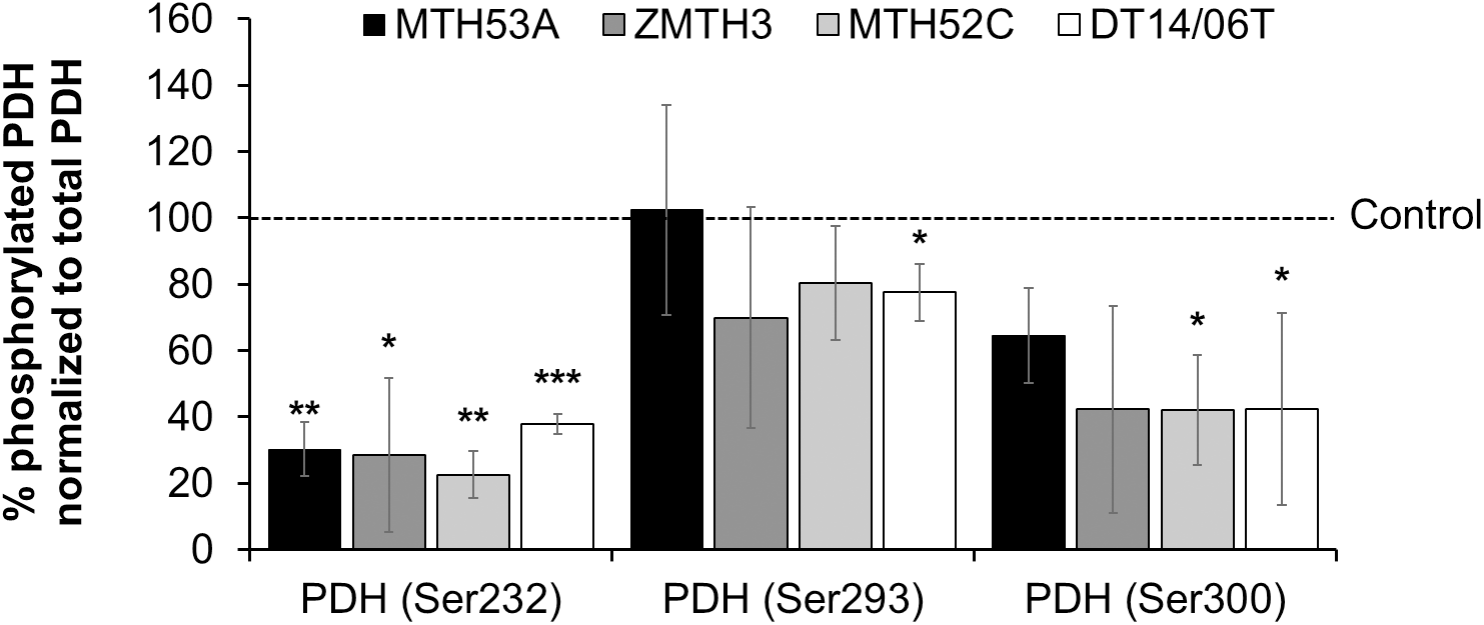
Effect of 10 mM DCA on PDH phosphorylation at three different residues after 48 hours treatment. Data was assessed with Luminex Magnetic Bead technology. PDH-P at Ser^232^ decreased significantly in all cell lines. Residue Ser^293^ showed no significant response to DCA treatment except in cell line DT14/06T. The third phosphorylation site Ser^300^ has decreased values in carcinoma cell lines but not in non-neoplastic mammary gland derived cell line MTH53A and benign cell line ZMTH3. Data are shown as mean ± SD, n≥3 and are presented as relative PDH-P values in comparison to untreated control (%). Control was set to 100%. Statistical analysis was performed with two-tailed t-test, *p<0.05, **p<0.01, ***p<0.001.

Phosphorylated PDH (PDH-P) and thus inactive PDH was measured with Luminex Magnetic Bead technology to evaluate whether DCA has an effect on pyruvate oxidation. After normalization to total PDH expression phosphorylated PDH at Ser^232^ decreased significantly in all cell lines whereas PDH-P at Ser^293^ was reduced only in the mammary carcinoma DT14/06T (p = 0.0445) (Fig 5). The mammary adenoma cell line ZMTH3 and mammary carcinoma cell line MTH52C showed apparent decreased average values, but due to variations between experiments no significance was observed. MTH53A showed no reduction of PDH-P at Ser^293^ in comparison to untreated control. With consideration of PDH-P at Ser^300^ a significant decrease is apparent between mammary carcinoma derived cell lines MTH52C (p = 0.0263), DT14/06T (p = 0.0282) and non-treated control. ZMTH3 showed visible tendency of PDH-P reduction but did not reach significance (p = 0.0852). No reduction was apparent in all PDH-P residues between MTH53A and neoplastic cell lines.

### DCA affects mitochondrial respiration

**Fig 6:**
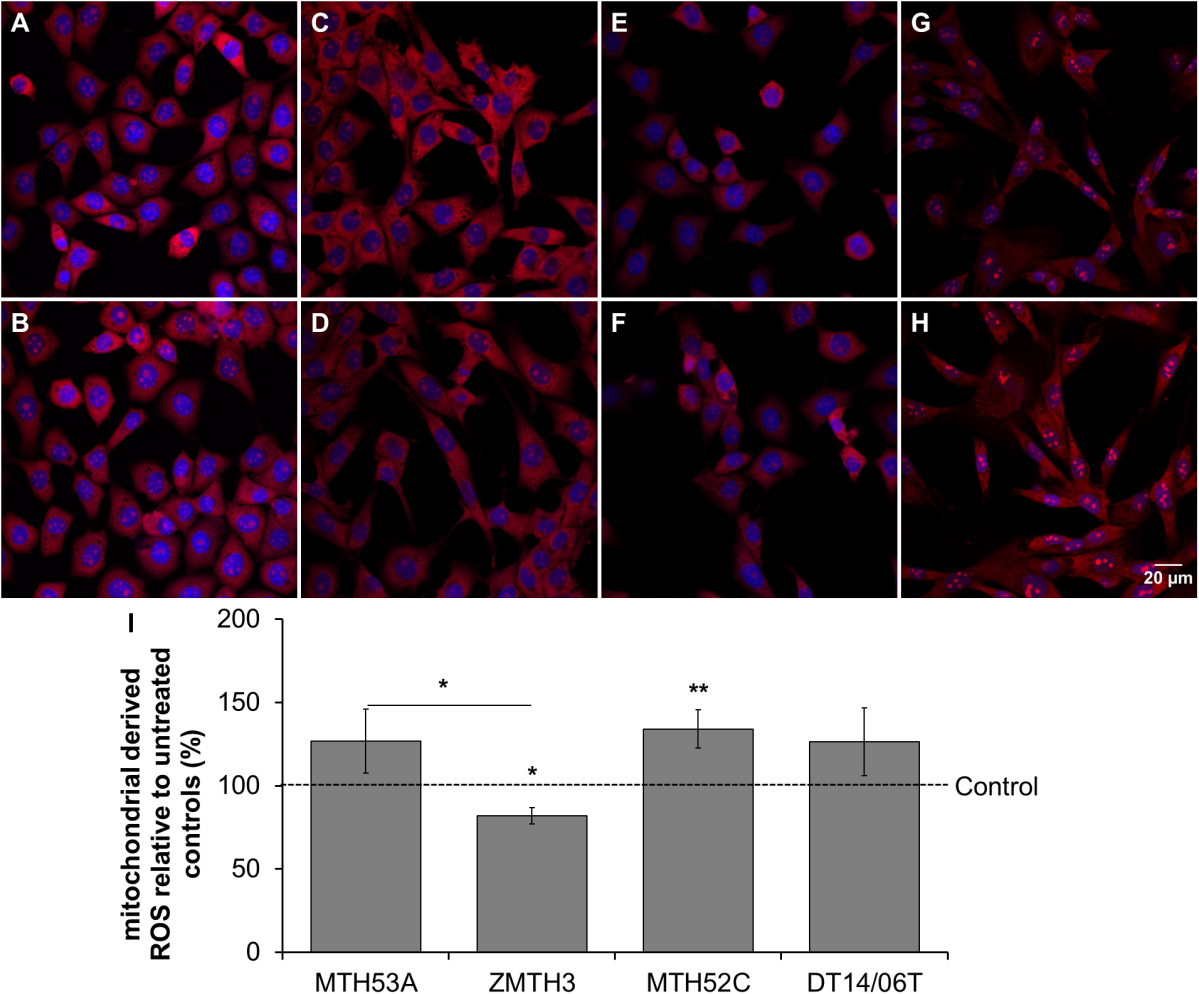
Influence of 10 mM DCA on mitochondrial activity in mammary cell lines after 48 hours. (A-H) Fluorescence of mitochondrial derived ROS (red) and counterstained cell nuclei (DAPI, blue). (I) In comparison to untreated control significant improvement of mitochondrial activity was observed in mammary carcinoma cell line MTH52C. A visible but insignificant increase was observable in the mammary carcinoma cell line DT14/06T and non-cancerous mammary gland derived cell line MTH53A. The adenoma cell line ZMTH3 showed significantly decreased mitochondrial activity in comparison to untreated control and non-cancerous cell line MTH53A. No significant difference was evaluated between MTH53A and the other cell lines. Data are shown as mean ± SD, n≥3 and are presented as relative fluorescence (mitochondrial activity) in comparison to untreated control (%). Control was set to 100%. Statistical analysis was performed with two-tailed t-test, *p<0.05, **p<0.01, ***p<0.001. (A) MTH53A control; (B) MTH53A+DCA; (C) ZMTH3 control; (D) ZMTH3+DCA; (E) MTH52C control; (F) MTH52C+DCA; (G) DT14/06T control; (H) DT14/06T+DCA; (I) relative mitochondrial activity.

**Fig 7:**
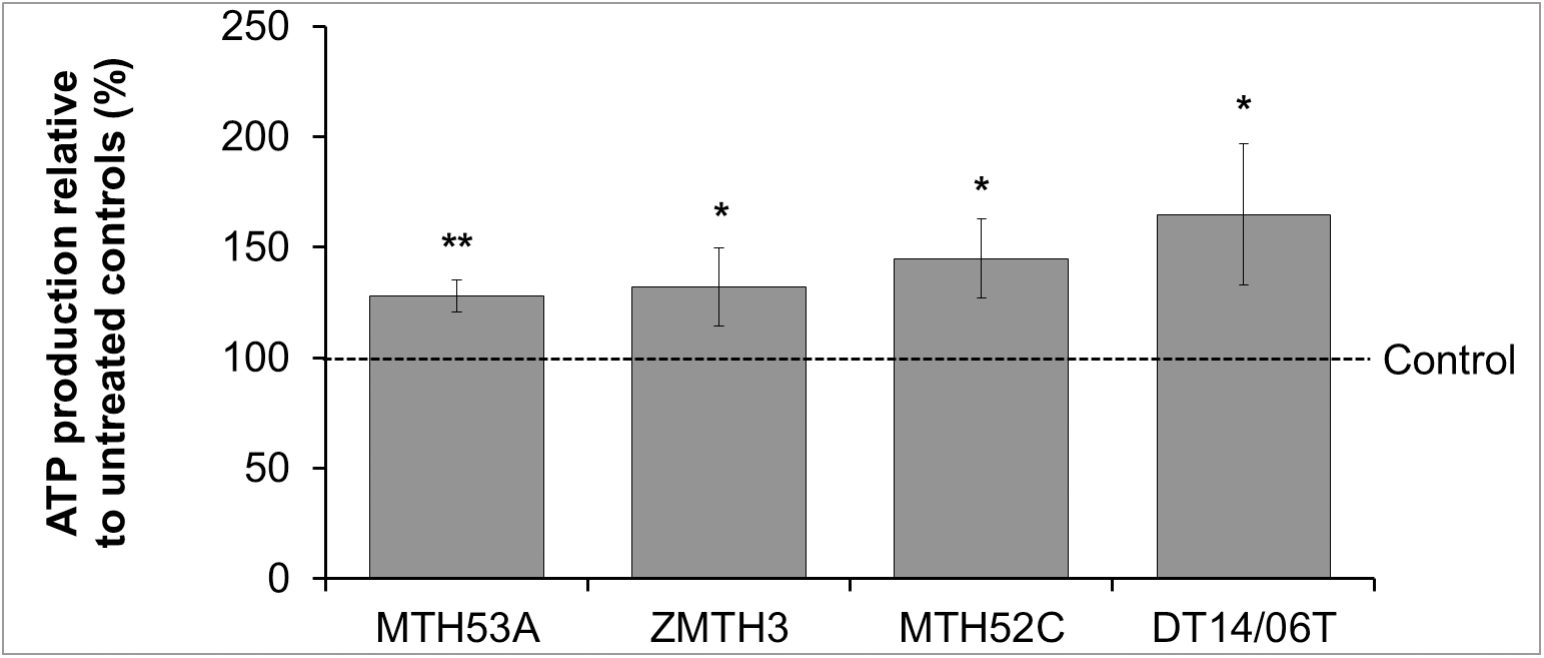
Influence of DCA on cellular ATP production in mammary cell lines after 48 hours. In comparison to untreated control significant enhancement of ATP-production was observed in all cell lines. No difference was detectable between non-neoplastic mammary gland derived cell line MTH53A and neoplastic tissue derived cell lines. Data are shown as mean ± SD, n≥3 and are presented as relative ATP-production (mitochondrial respiration) in comparison to untreated control (%). Control was set to 100%. Statistical analysis was performed with two-tailed t-test, *p<0.05, **p<0.01, ***p<0.001.

Mitochondrial respiration was evaluated via indirect measurement of mitochondrial derived ROS. As shown in Fig 6 the production of mitochondrial derived ROS increased slightly in MTH53A (p = 0.1386), DT14/06T (p = 0.1519) and significantly in MTH52C (p = 0.0359). In contrast the benign cell line ZMTH3 showed significantly decreased ROS production and thus decreased mitochondrial activity (p = 0.0243). A statistical significant difference is apparent by comparison of MTH53A and ZMTH3 (p = 0.0178). Additionally mitochondrial respiration was directly assessed by cellular ATP synthesis. A significant increased ATP production after DCA exposure in all cell lines was shown (Fig 7). No apparent difference was observed between the evaluated cell lines.

### DCA did not affect apoptosis

**Fig 8:**
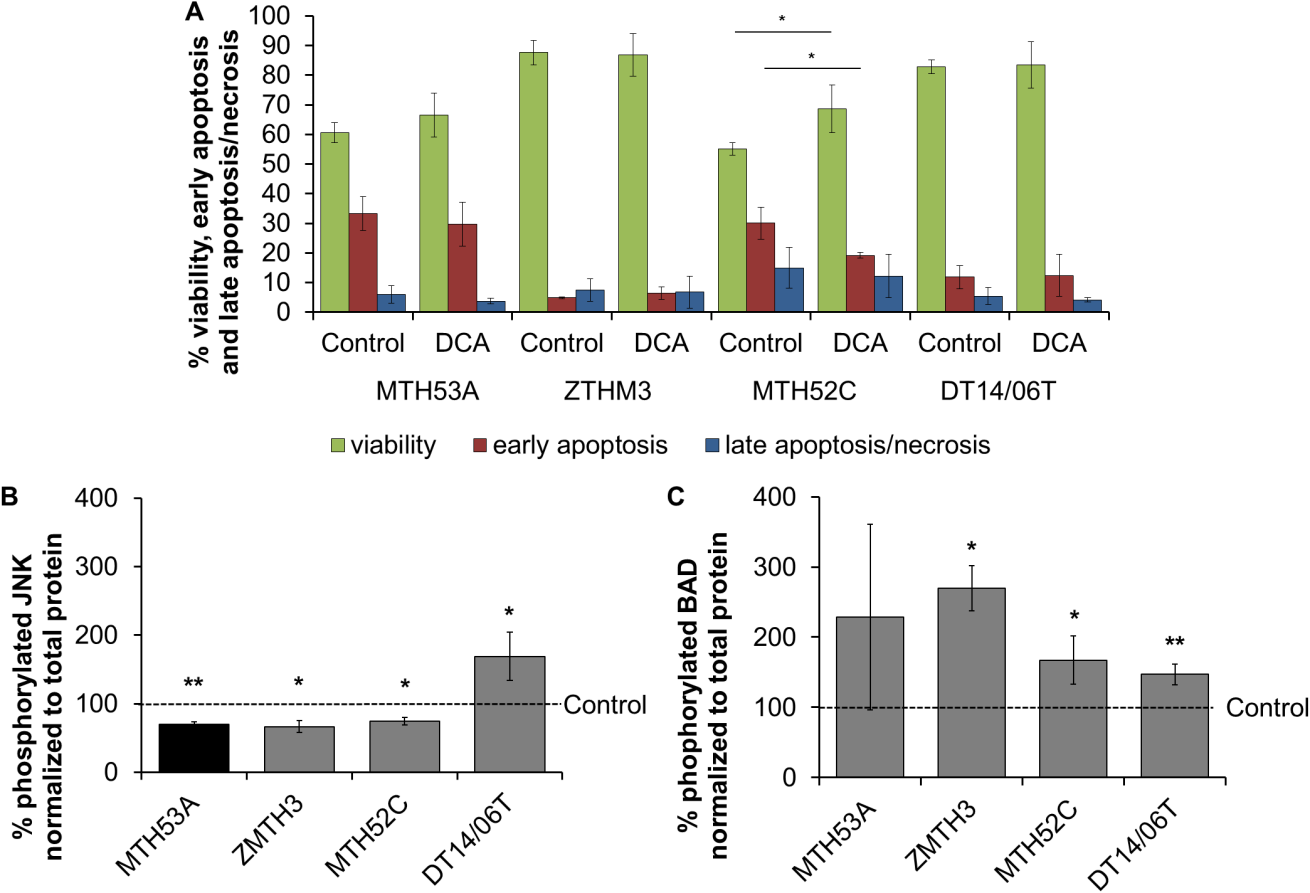
Effect of 10 mM DCA on protein expression in mammary cell lines after 48 hours. In comparison to negative control, significant decrease in JNK expression was observed in all cell lines except DT14/06T which showed increased JNK values. Significantly increased BAD expression was observed in all cell lines except MTH53A. Data are shown as mean ± SD, n≥3 and are presented as relative mean in comparison to untreated control (%). Control was set to 100%. Statistical analysis was performed with two-tailed t-test, *p<0.05, **p<0.01, ***p<0.001. (A) Apoptotic and dead cells in flow cytometry; (B) relative expression of JNK; (C) relative expression of BAD.

After 48 hours DCA treatment, viability analysis with flow cytometry revealed no changes in apoptosis and amount of dead cells in MTH53A, ZMTH3 and DT14/06T (Fig 8A). In MTH52C the percentage of apoptotic cells (p = 0.0256) decreased significantly after DCA exposure and accompanied with significantly increased viability (p = 0.0472).

To determine if trypsinization caused negative effects, apoptosis was evaluated with TUNEL method via fluorescence microscopy. No difference in number of TUNEL positive cells was evaluated in any of these cell lines (S1 Fig).

Further, two proteins (c-jun N-terminal kinases and Bcl-2-Antagonist-of-cell-death) involved in apoptosis were evaluated. The protein expression was performed with Luminex Magnetic Bead technology. After 48 hours of DCA treatment, the phosphorylated and thus activated c-jun N-terminated kinases (JNK) decreased significantly in MTH53A (p = 0.0046), ZMTH3 (p = 0.0228) and MTH52C (p = 0.0131). The inverse case was observed in mammary carcinoma cell line DT14/06T (p = 0.0121) presenting significantly increased active JNK expression (Fig 8B).

The phosphorylated and thus inactive form of Bcl-2-Antagonist-of-cell-death (BAD) showed significantly increased values in all neoplastic cell lines. In MTH53A (p = 0.2340) no difference was detectable (Fig 8C).

### DCA decreased survivin expression

**Fig 9:**
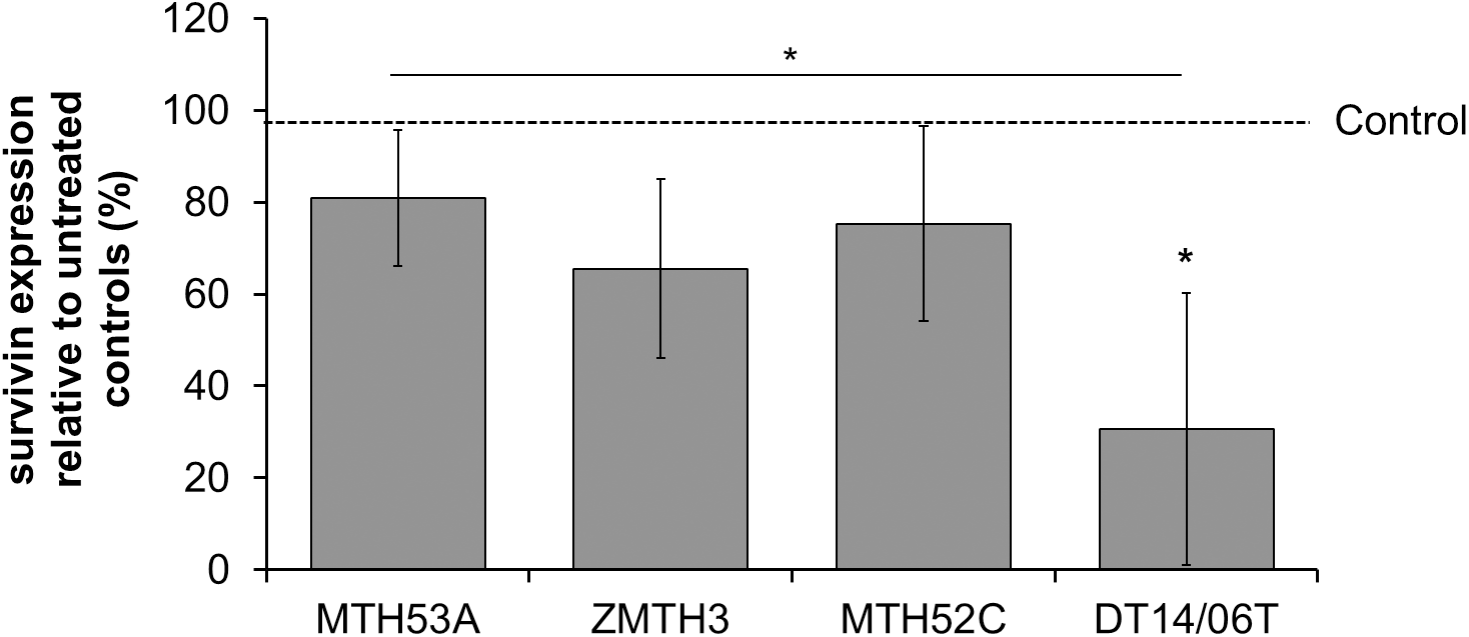
Effect of 10 mM DCA on survivin production in several mammary cell lines after 48 hours. Significant reduction of survivin expression was observed in DT14/06T in comparison to untreated control and MTH53A. Data are shown as mean ± SD, n = 3 and are presented as relative survivin expression in comparison to untreated control (%). Control was set to 100%. Statistical analysis was performed with two-tailed t-test, *p<0.05, **p<0.01, ***p<0.001.

Decreased survivin expression is apparent in all cell lines but significance is present in cell line DT14/06T (p = 0.0125) in comparison to untreated control as well as in comparison to non-neoplastic mammary gland derived cell line MTH53A (p = 0.0200) (Fig 9).

### Metabolic activity following 96 hours continuous DCA treatment

**Fig 10:**
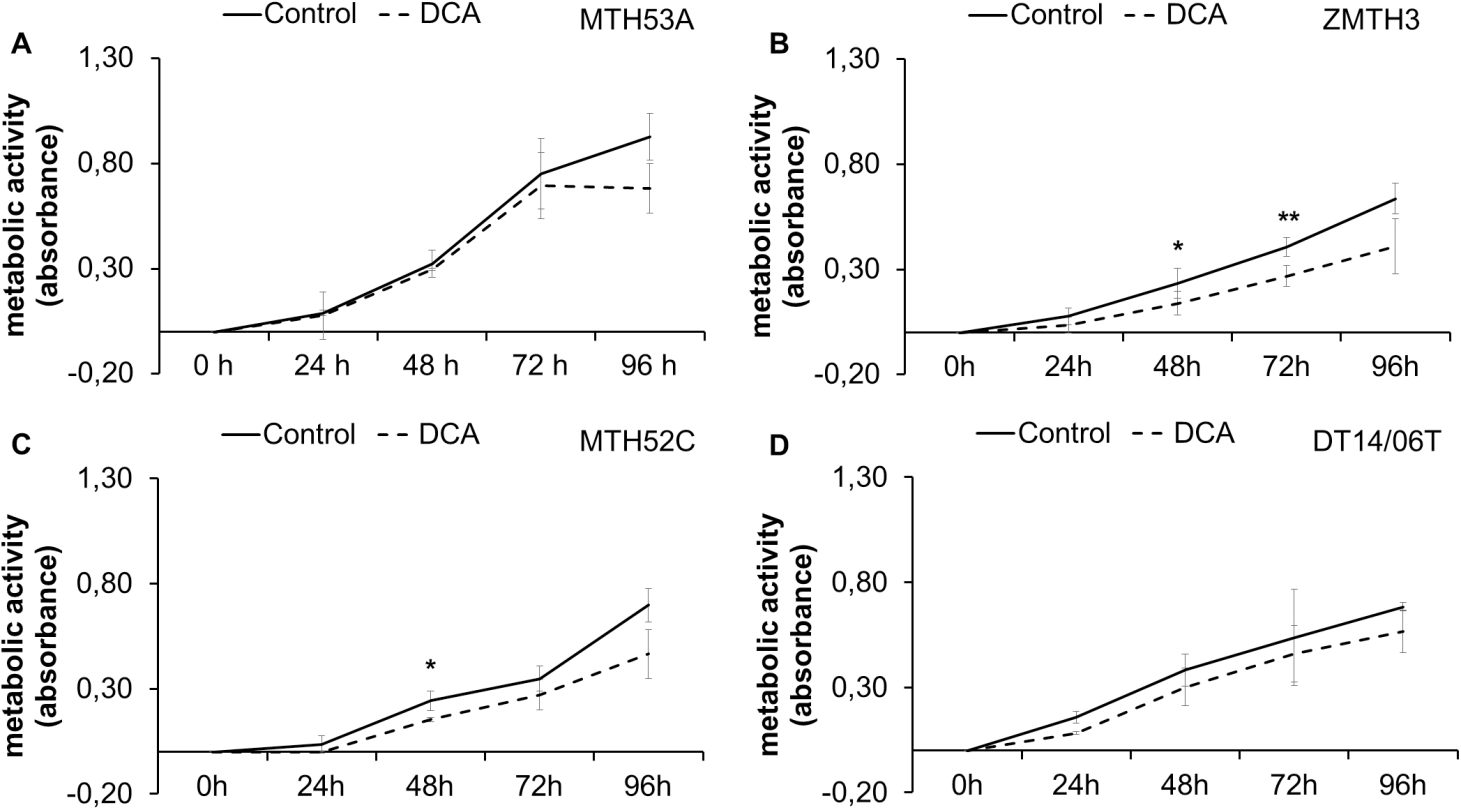
Effect of 10 mM DCA on metabolic activity after 48 hours. In comparison to untreated control no significant reduction metabolic activity was observed in MTH53A (A) and DT14/06T (D). ZMTH3 (B) showed significantly reduced metabolic activity after 48 hours and MTH52C after 24 hours (C). Data are shown as mean ± SD, n = 3 and are presented as relative metabolic activity in comparison to untreated control (%). Control was set to 100%. Statistical analysis was performed with two-tailed t-test, *p<0.05, **p<0.01, ***p<0.001. (A) MTH53A; (B) ZMTH3; (C) MTH52C; (D) DT14/06T.

The metabolic activity as reference for proliferation and viability was assessed during 96 hours ongoing DCA treatment (Fig 10). After 96 hours the non-neoplastic cell line MTH53A and the mammary carcinoma cell line DT14/06T showed no significant differences in metabolic activity compared to untreated control. Merely ZMTH3 showed significant decreased metabolic activity starting at 24 hours whereas MTH52C displayed significantly decreased MTT-values after 24 hours. Following DCA treatment metabolic activity in MTH52C converge to non-treated control.

### MicroRNA expression after DCA exposure

**Fig 11:**
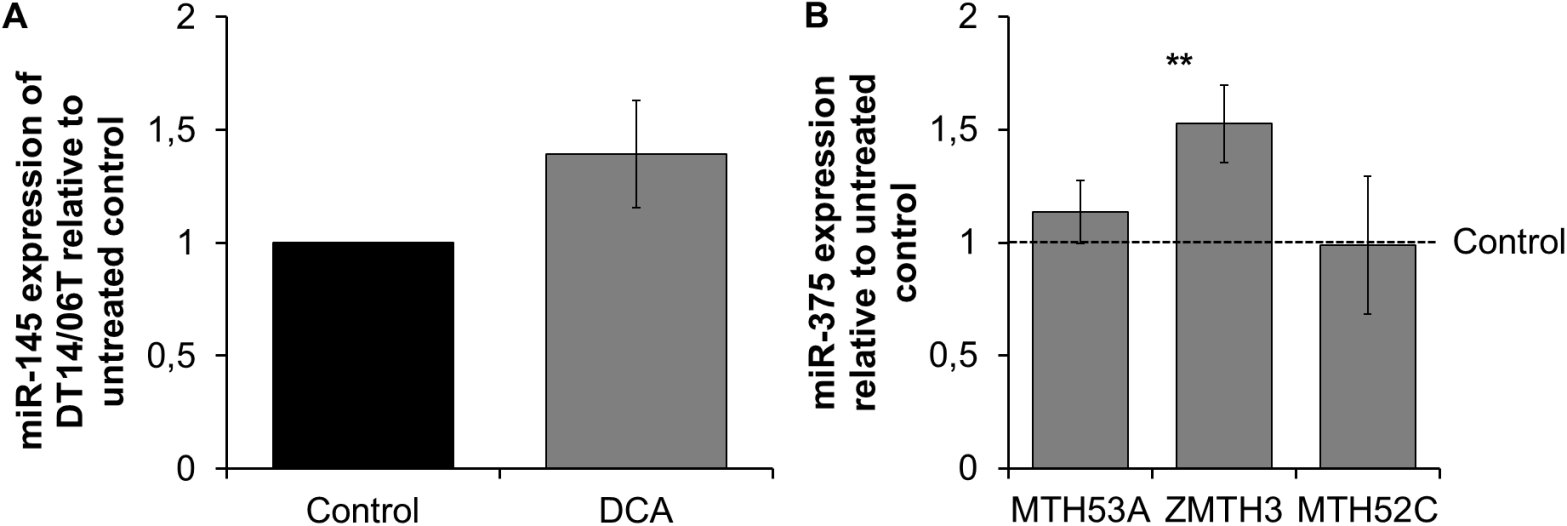
Effect of 10 mM DCA on miR expression after 48 hours. In comparison to untreated control no significant changes in miR-145 was proved. Regarding miR-375, a significant increase was observed in ZMTH3. Data are shown as mean ± SD, n = 3 and are presented as relative miR expression in comparison to untreated control. Ct-values were analyzed with Rest2009 and statistical analysis was performed with two-tailed t-test, *p<0.05, **p<0.01, ***p<0.001. (A) miR-145 expression of DT14/06T; (B) miR-375 expression.

Three microRNAs involved in apoptosis and proliferation were analyzed with quantitative RT-PCR. miR-145 was detectable only in the mammary carcinoma cell line DT14/06T (Fig 11A) and showed no significant difference after DCA treatment (p = 0.1025), the other cell lines showed basal expression under limit of detection and were excluded from analysis. Regarding miR-375, significant changes after DCA treatment revealed in the cell line ZMTH3 (p = 0.0086), whereas MTH53A and MTH52C showed no modifications in miR expression (Fig 11B). miR-141 expression was below the limit of detection in all cell lines and was excluded from analysis due to Ct-values >35.

## Discussion

The initial aim of this study was to determine which metabolic processes occur in canine mammary gland derived cell lines during DCA exposure and if DCA might be beneficial for cancer treatment in dogs bearing mammary gland neoplasia. The results presented illustrate that DCA has anti-proliferative effects on cell growth which is shown in decreased cell number and reduction of the proliferation marker Ki67 expression. The growth inhibiting effect was likewise observed in several human studies [29–32] including mammary neoplastic and non-neoplastic cell lines [11, 18, 33] as well as in canine prostate and bladder cancer cell lines [20]. The decreased expression of Ki67 indicates a delayed cell division resulting in changes of expression of several proteins involved in differentiation, as described by Vella et al. [34]. The verification of the proteomic changes leading to the observed alteration cell division would require broad proteome analysis. In comparison to canine prostate adenocarcinoma and transitional cell carcinoma cell lines [20], the mammary gland derived cell lines showed a decreased responsiveness to DCA compared to transitional cell carcinoma cell lines. However, the observed response was similar to effects reported for canine prostate adenocarcinoma cell lines. In contrast to human cell lines, no significant difference was observed between the non-neoplastic mammary gland derived cell line and neoplastic cell lines [11, 18, 35].

Due to increased cellular respiration and thus mitochondrial ROS production, it is hypothesized that DCA induces mitochondrial dependent apoptosis by release of cytochrome c and activation of several apoptosis proteins [11]. However, the mammary carcinoma cell line MTH52C showed increased mitochondrial derived ROS production but no increase in apoptosis was observed in the examined cell lines. Instead a decreased apoptosis and increased viability after DCA treatment was observed in MTH52C. This result is similar to observations in human mammary and neuroblastoma cell lines [17]. The observed effect could be a result of protective hormesis a phenomenon reported to describe the effect of low dosages of toxic substances resulting in higher viability [36]. Further it should be noted that not all ROS are efficient enough to induce apoptosis [37]. Despite ROS increase, it might be possible that not enough apoptosis inducing ROS mediators, e. g. hydrogen peroxide or hypochloric acid [37], are produced during DCA treatment. Significant decreased values in active JNK protein were observed in all cell lines with exception of DT14/06T which showed significantly increased activated JNK protein. Also BAD, which was determined as inactivated protein increased significantly in all cell lines. Merely MTH53A showed no effect after DCA treatment. The increased active form of JNK in the mammary carcinoma cell line DT14/06T could not be verified by flow cytometry and TUNEL method. Therefore it has to be suggested that active JNK, a cellular kinase with multiple targets and effects [38], is possibly not sufficient for apoptosis induction or has other non-apoptotic but transcriptional functions in this cell line. The metabolic activity, as indicator for cell viability, did not indicate a decrease of values in all cell lines after 96 hours and is consistent with data provided from flow cytometry. Stockwin et al. reported that high concentrations of DCA are required for apoptosis induction [19]. The same extent was noticed in canine prostate adenocarcinoma and transitional cell carcinoma cell lines [20] and several human cancer entities as well as in mammary cancer using dosages between 0.5 and 40 mM DCA [17, 18, 35, 37, 39–41], whereas Bonnet et al. was able to induce apoptosis with 0.5 mM DCA in human MCF-7 cells. Further Babu et al. reported that SLC5A8 transporters are required for membrane transportation of DCA [35]. SLC5A8 is silenced in many cancer tissues as well as in mammary cancer, so that DCA might not accumulate in mammalian cells and apoptosis was not induced with 1 mM DCA [35]. The same cancer cell lines expressing SLC5A8 transporters undergo apoptosis when treated with 1 mM DCA [35]. Different SLC5A8 expression in cancer cell lines would declare why apoptosis is apparent inconsistently.

Apart from this, survivin expression, an apoptosis inhibitor and proliferation promotor [42, 43], showed slight but insignificant decreasing values (with exception of the mammary carcinoma derived cell line DT14/06T) following DCA treatment and supports the hypothesis that DCA has no pro-apoptotic effects on canine mammary cancer cells. These results are divergent to canine prostate adenocarcinoma and transitional cell carcinoma cell lines, which showed reduced survivin expression. Nevertheless a supportive anti-proliferative effect in mammary gland derived cell lines might be possible. In case of the cell line DT14/06T the decreased survivin expression might have anti-proliferating effect but seems to be too low to induce apoptosis.

DCA, a known PDK inhibitor, shifted the energy production from glycolysis towards normal cellular respiration with glucose oxidation [9]. This is illustrated by decreased lactate release, reduction in PDH phosphorylation and increased ATP-production and mitochondrial respiration, respectively. The results suggest that PDH phosphorylation at Ser^232^ and Ser^300^ appear to be the most important metabolic modulation in canine PDH regulation. This supports the assumption of Rardin et al., that PDH phosphorylation at Ser^232^ is not limited to heart tissue [44]. In contrast to studies in humans, Ser^293^ phosphorylation was not significantly affected by DCA [29, 30]. Ser^293^ is reported to be phosphorylated rapidly by PDK, especially isoform 2 [44], indicating that DCA might not influence all PDK isoforms equally in dogs. The data provided demonstrate a shift towards normal cellular respiration which might declare the anti-proliferative effect. Further the PDK-1 and PDK-3 expression was also analyzed to evaluate, if changes in PDH phosphorylation were caused by decreased PDK expression. As the provided data show no changes neither in PDK-1 nor in PDK-3 expression was observed. This data supports the findings, that DCA indirectly activates PDH and thus promotes cellular respiration towards glucose oxidation. Due a lack of canine specific PDK-2 and PDK-4 antibodies, no statement regarding these two isoforms can be drawn. A different expression of PDK-2 or PDK-4 due to DCA treatment appears to be improbable.

In order to evaluate the effect of DCA on cell division and apoptosis, miR-141, miR-145 and miR-375, often deregulated in cancer [45–47], were analyzed. MiR-145 is reported to be a tumor suppressor and usually downregulated in mammary breast cancer tissues [48, 49]. The used dose of DCA had no effect on miR-145 expression neither in benign nor in malign mammary tumors. Thus, a general miR-145 effect in the herein analyzed canine mammary tissue derived cell lines appears unlikely. MiR-375 was significantly upregulated (1.5 fold) in mammary adenoma cell line, which may indicate that proliferation may be influenced by miR-375. It has to be considered, that this can also occur due to biological variations within experiments. Ward et al. showed, that upregulation of miR-375 could sensitize mammary cancer cells to Tamoxifen [50]. Nevertheless, Tamoxifen was reported to induce adverse effects in dogs [51], a sensitization to other cytotoxic components might be beneficial, if DCA is able to alter miR-375 expression. For miR-141, no changes were observed due to basal expression under limit of detection and exclusion from analysis.

In comparison to human mammary cancer cell lines following DCA treatment, canine mammary cell lines are almost consistent [18, 41]. DCA has anti-proliferative effects on canine mammary neoplasia cell lines including non-neoplastic mammary gland derived cells, but no apoptosis inducing properties. Further glycolytic property was inverted towards glucose oxidation as shown in human mammary cancer [11]. In contrast to canine prostate adenocarcinoma and transitional cell carcinoma cell lines, cell lines derived from mammary gland tissue showed also an anti-proliferative effect with no apoptosis induction. However, these cell lines appear to be more resistant regarding cell number and proliferation, survivin expression and miR-expression. Merely changes in lactate production, PDH phosphorylation and thus cell respiration are comparable to both canine cancer entities examined by Harting et al. [20].

Further the dose of 10 mM DCA is clinically not achievable *in vivo* without severe side effects. But these data indicate that DCA might be supportive in sensitization of cancer cells to other chemotherapeutics [52]. Further a post-surgical therapy with drug releasing inlays implanted in the surgical area could be beneficial to achieve higher local concentrations of DCA to treat remaining cancer cells [53].

## Supporting information

S1 Fig Percentage of TUNEL positive cells (apoptotic) after DCA exposure determined with fluorescence microscopy. In comparison to non-treated control, no changes in apoptosis were observed. Data are shown as mean ± SD; n = 3 and are presented as percentage of TUNEL positive cells in comparison to negative control. Statistical analysis was performed with two-tailed t-test; *p>0.05.
(TIFF)

S2 Fig Effect of 10 mM DCA on PDK-3 expression after 48 hours. Western Blot analysis of PDK-3 expression in MTH53A, MTH52C, ZMTH3 and DT14/06T. PDK-3 expression was not detectable in all cell lines. GAPDH and α-tubulin were used as loading control.
(TIFF)
