# Supplementary figures and images for: Dichloroacetate affects proliferation but not apoptosis in canine mammary cell lines
Source: PLoS One. 2017 Jun 7;12(6):e0178744. doi: 10.1371/journal.pone.0178744 (PMC5462399; doi:10.1371/journal.pone.0178744)

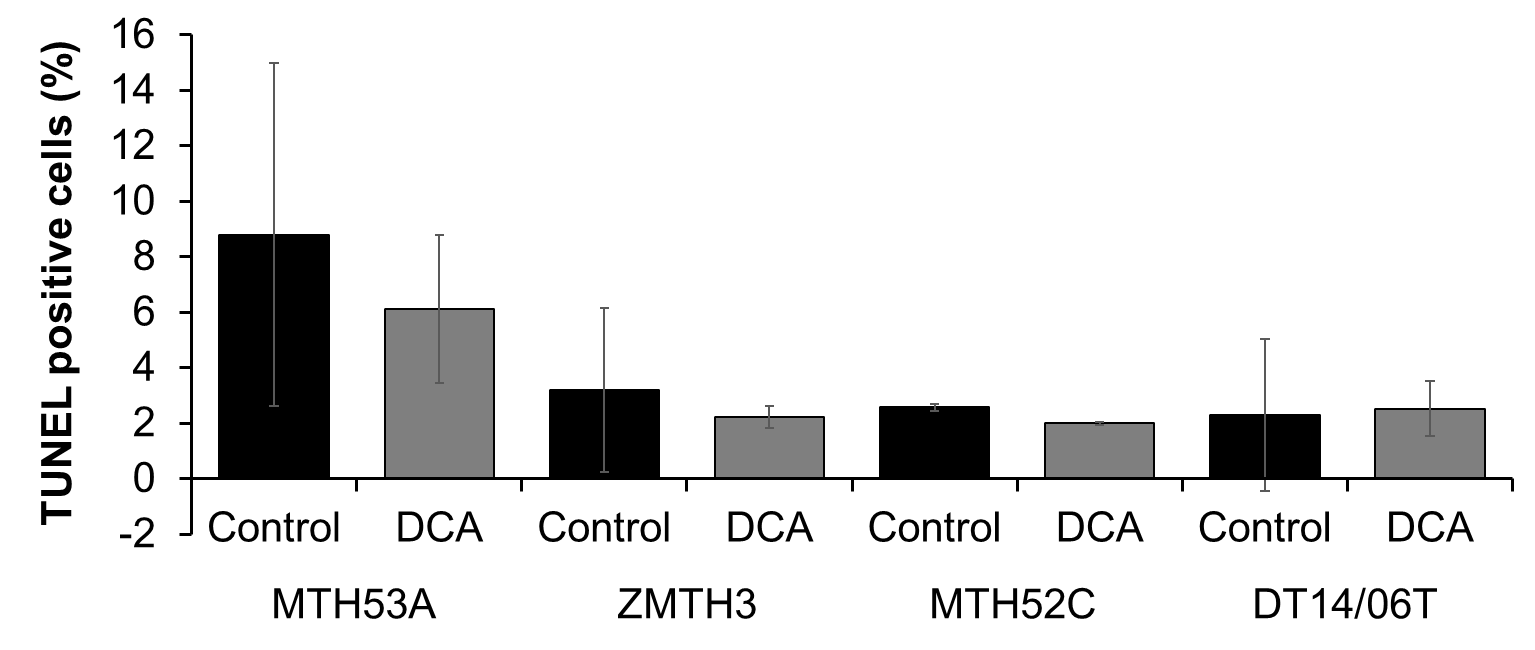

Supplement: S1 Fig — In comparison to non-treated control, no changes in apoptosis were observed. Data are shown as mean ± SD; n = 3 and are presented as percentage of TUNEL positive cells in comparison to negative control. Statistical analysis was performed with two-tailed t-test; *p>0.05. (TIFF) [file pone.0178744.s001.tiff]

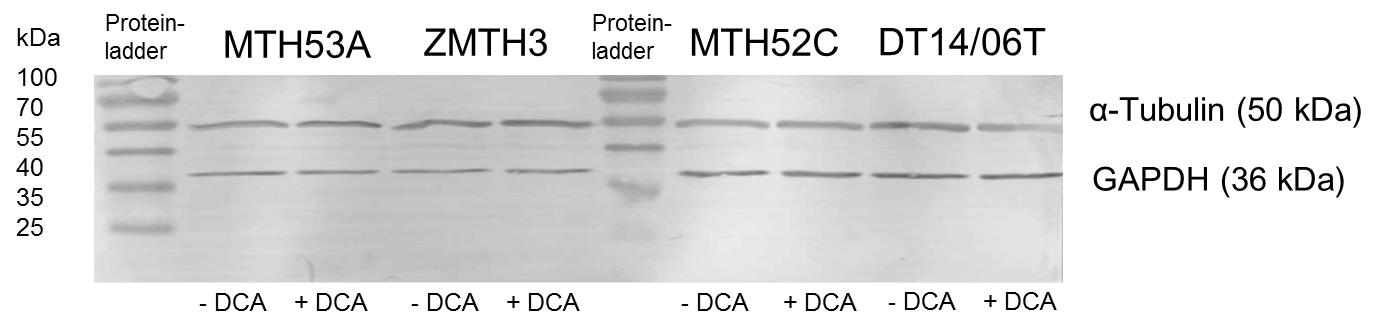

Supplement: S2 Fig — Western Blot analysis of PDK-3 expression in MTH53A, MTH52C, ZMTH3 and DT14/06T. PDK-3 expression was not detectable in all cell lines. GAPDH and α-tubulin were used as loading control. (TIFF) [file pone.0178744.s002.tiff]
